# Changes in Neuroimmunological Synapses During Cerebral Ischemia

**DOI:** 10.1007/s12975-024-01286-1

**Published:** 2024-08-05

**Authors:** Lynn Bitar, Berta Puig, Thomas G. Oertner, Ádám Dénes, Tim Magnus

**Affiliations:** 1 Neurology Department, Experimental Research in Stroke and Inflammation (ERSI) Group, University Medical Center Hamburg-Eppendorf (UKE), Martinistraße, 52, Hamburg, 20246 Germany; 2 Institute for Synaptic Physiology, Center for Molecular Neurobiology (ZMNH), University Medical Center Hamburg-Eppendorf, Hamburg, Germany; 3 “Momentum” Laboratory of Neuroimmunology, Institute of Experimental Medicine, Budapest, Hungary

**Keywords:** Neuroimmune synapse, Ischemic stroke, Innate immune system, Adaptive immune system

## Abstract

The direct interplay between the immune and nervous systems is now well established. Within the brain, these interactions take place between neurons and resident glial cells, i.e., microglia and astrocytes, or infiltrating immune cells, influenced by systemic factors. A special form of physical cell–cell interactions is the so-called “neuroimmunological (NI) synapse.” There is compelling evidence that the same signaling pathways that regulate inflammatory responses to injury or ischemia also play potent roles in brain development, plasticity, and function. Proper synaptic wiring is as important during development as it is during disease states, as it is necessary for activity-dependent refinement of neuronal circuits. Since the process of forming synaptic connections in the brain is highly dynamic, with constant changes in strength and connectivity, the immune component is perfectly suited for the regulatory task as it is in constant turnover. Many cellular and molecular players in this interaction remain to be uncovered, especially in pathological states. In this review, we discuss and propose possible communication hubs between components of the adaptive and innate immune systems and the synaptic element in ischemic stroke pathology.

## Background

Synaptic plasticity is the activity-dependent modification of the synaptic transmission at preexisting synapses, which in adult neural circuits is a dynamic process involving both strengthening and weakening of existing synapses [1, 2]. Many forms and mechanisms of plasticity have been described given its diverse functions [2]. It is associated with the remodeling of dendritic spines, including increased spine volume, stability, and clustering [3]. A key structural change influencing plasticity is axonal sprouting which could be involved in rewiring of neural circuits [4–7]. Pruning is another process necessary for the regulation and promotion of synapse formation [8]. Plasticity occurs at the level of individual synapses that connect neurons and influence the neuronal network. However, the various resident and nonresident cell types in the brain affect neural circuits through either direct or indirect interactions with neurons [9]. Synapses are shaped by various intercellular interactions involving neurons, immune cells, epithelial cells, and even pathogens and host cells [10]. Despite the highly organized molecular structure of synapses, they require constant extrinsic input and respond to a host of secreted factors [11]. This transsynaptic signaling dictates various aspects of synaptic signaling, specificity, and stability in homeostasis and especially in disease [11, 12]. Communication between neurons and other cell types occurs at multiple levels, in part by diffusing molecules that reach their target receptors and activate downstream signaling pathways [13]. These receptors can be located at or near synapses, thus directly modulating synaptic functions, which is ultimately critical for the coherence and behavior of neuronal networks [14–17].

In cerebral ischemia, the transient or permanent lack of blood flow leads to synaptic failure in neurons that are directly supplied by the affected vessels but also spreads to the surrounding tissue through various mechanisms such as a dysfunction of ligand- and voltage-gated channels or disrupted docking of glutamate-containing vesicles [36, 37]. Concomitantly, both resident glial cells in the parenchyma and peripheral immune cells markedly change their activity in response to cerebral ischemia, resulting in the recruitment of immune cells to the injured area [38]. These immune responses lead to structural and functional loss of synapses, followed by rearrangement of neuronal networks, axonal sprouting, and remyelination [38]. Consequently, defective synaptic-immune interactions are likely to lead to a homeostatic imbalance between glial or immune cells and neurons at synaptic sites that disrupts proper neuronal communication and contribute to the ischemic stroke progression or impaired recovery [18].

The anatomical connection forming these synaptic-immune interactions extends to functional consequences which constitutes the previously described neuroimmunological synapse [19, 20]. It refers to specialized zones of interaction between neural and immune entities that assert physiological roles in synaptic transmission and plasticity or may initiate, promote, and sustain pathological states in the brain [18]. To understand the pathophysiology of diseases like cerebral ischemia, it is necessary to understand how NI synapses function, as they dictate neuronal network activity and promote or sustain pathological states. In this way, immune cells are able to affect neural function within the network. In this review, we discuss crosstalk between synapses and immune cells of both the adaptive and innate systems that may influence stroke outcome. We report direct interactions between a lymphocyte/intraparenchymal APC and neuronal synapses, reflecting a “classical” neuroimmune synapse, or indirect communication via immune-secreted factors that directly affect synaptic function.

## The NI Synapses

**Fig. 1:**
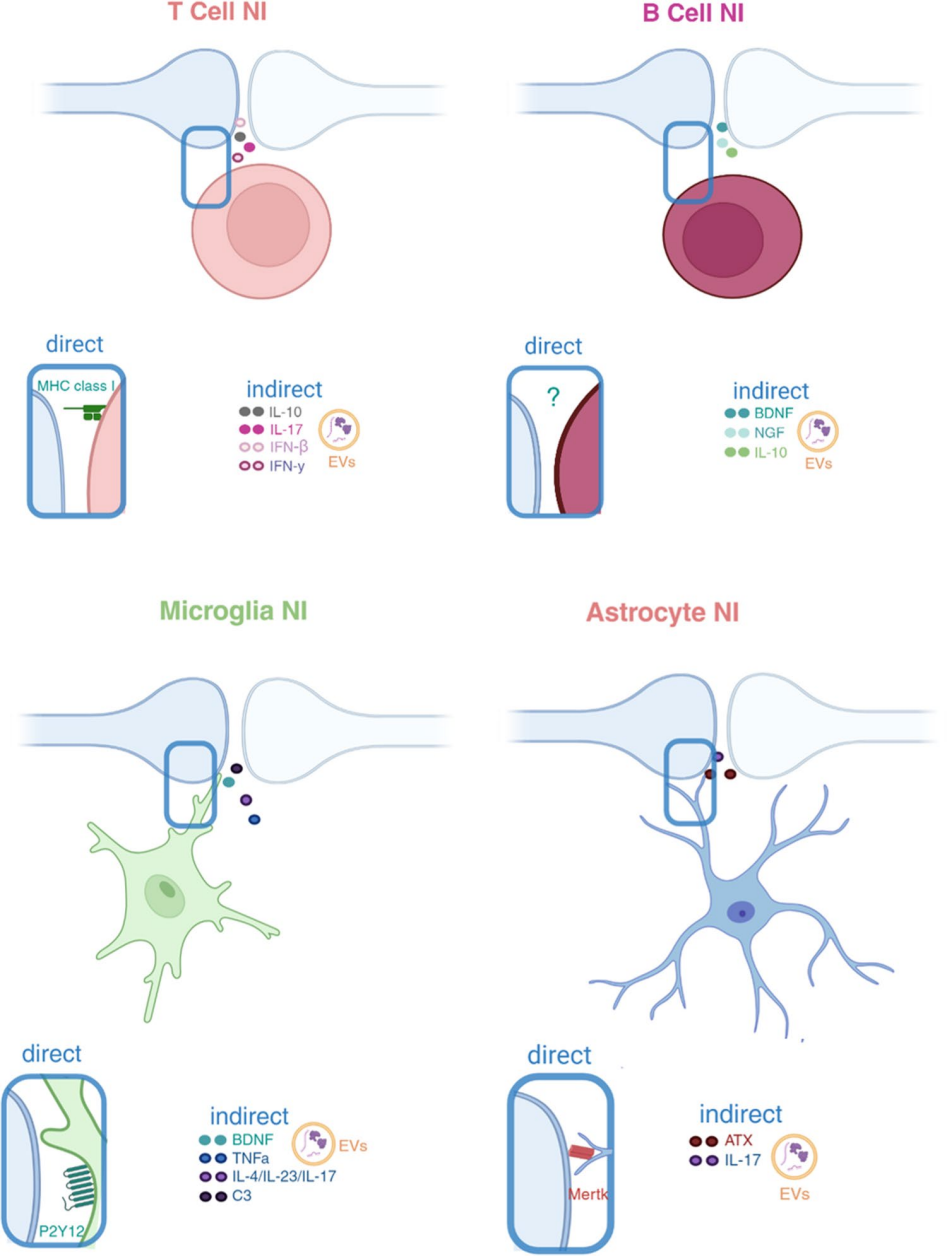
Overview of direct and indirect interactions of adaptive and innate immune cells within the NI synapse in an ischemic context. The precise interaction sites are not yet fully elucidated. This scheme is a general representation.  MHCI, major histocompatibility complex; IL, interleukin; EVs, extracellular vesicles; BDNF, brain-derived neurotrophic factor; NGF, nerve growth factor; TNF-*α*, tumor necrosis factor alpha; C3, complement; ATX, autotaxin. All figures were created using BioRender

**Fig. 2:**
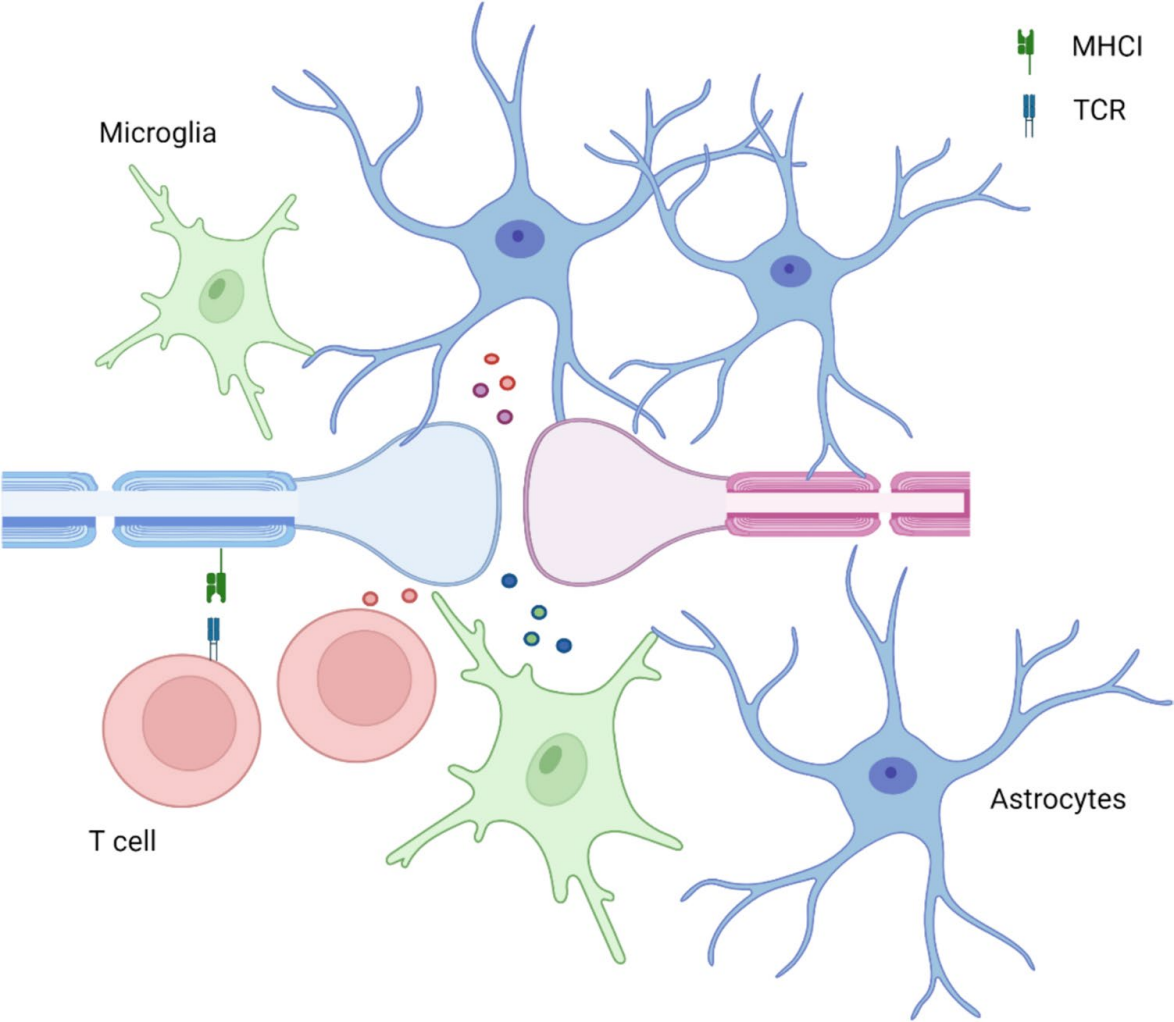
Schematic figure showing a quadripartite synapse along with potential site of T-cell interaction with synapses

Bidirectional communication between neurons and immune cells has been reported in the nervous system [21] (Fig. 1). It has also been described that elements of the immune system, including T cells and macrophages, but also microglia and astrocytes, influence synaptic transmission and intrinsic excitability (Fig. 2) [22–26]. Recently, the existence of functional interactions between synapses and inflammatory or immune mediators in the central nervous system (CNS) has been highlighted, showing an influence on both pathological and physiological states [27–32]. More recently, the presence of microglial-synaptic contacts in the CNS has been described, which may be associated with several autoimmune or neurodegenerative diseases and injuries [33, 34]. Specifically, during inflammation, glia and neurons may operate as competent antigen-presenting cells (APCs) that process and present antigens via major histocompatibility complex (MHC) molecules [35–37]. Evidence from developmental studies revealed that the localization of multiple immune components at synapses helps fine-tune synapse formation or pruning and thus control neuronal networks [38]. Based on this, the term NI synapse was first used to describe specialized contact zones with functional and molecular connections between adrenergic neurons and lymphocytes or antigen-presenting cells (APCs) [19]. Immunological synapses (IS), the specialized junction between APCs and T cells during T-cell activation, were described more than 40 years ago [39, 40]. However, NI synapses are more closely related to neuronal synapses than to IS. Both systems have evolved to have specific molecular recognition events between distinct cells, positional stability, organized secretion for communication, and sophisticated forms of information storage [19, 41]. Neuro-immune interactions rely on contact zones to directly transmit and transduce highly controlled secretory signals, generally mediated by soluble factors such as neurotransmitters, neuropeptides, or cytokines [41, 42]. In the case of IS, membrane protein interactions on opposing surfaces of cellular membranes trigger intracellular signaling cascades that allow precisely controlled communication between different cells. Key elements of these interactions, including the reorganization of signaling proteins at the contacts, can even be modeled using synthetic membranes or vesicles. To control different biological processes in the CNS, similar forms of membrane-membrane communication exist between neurons via synaptic membranes or gap junctions, within the syncytial network of astrocytes, between astrocyte endfeet and synapses or endothelial cells, and between microglial processes or cell bodies and different neuronal compartments [43]. Considering the heterogeneity of the cellular composition of the CNS at different sites, the different origin of glial cells (i.e., neuroectodermal astrocytes vs mesodermal microglia), and the surveillance of brain tissue by immune cells under physiological conditions or in pathological states, possibilities for cell–cell interactions are vast and complex and have not yet been efficiently categorized. However, there are some features that distinguish NI synapses from classical neuronal synapses. The first is the cellular components involved, i.e., in this case, a synapse forming between a cellular compartment of a neuron and an “immune cell.” Second, NI synapses have synaptic clefts that are only 6 nm wide as opposed to 15–30 nm in IS, leading to higher intracleft neurotransmitter concentrations which enhance the activation of synaptic receptors and allow for more efficient synaptic transmission [19, 44]. Third, the postsynaptic cells such as lymphocytes or macrophages in neuroimmune synapses secrete cytokines that enable a secondary mode of communication through feedback signals [19]. Retrograde signaling by secretion from the postsynaptic element also occurs at some neuronal synapses, however secreted factors are different (e.g., endocannabinoids, BDNF) [45, 46].

## Neuro(adaptive)immunological Synapses with Direct Neuronal Effects

Adaptive immune cells such as T and B cells and their mediators influence the function of the nervous system and participate in plasticity and synapse elimination during development as well as in the adult steady state [47]. Although the entry of immune cells into the brain parenchyma is relatively limited in healthy individuals, they constantly circulate in the meningeal lymphatic vessels [48–55]. This migratory step is crucial for immune surveillance as they continuously scan the local environment for cognate antigens [56–58]. This is further supported by findings that the absence of these cell types can impair cognitive function, affect spatial learning [59, 60], and reduce neurogenesis in the dentate gyrus of murine hippocampus [61]. In early studies by Kipnis and colleagues, they showed that mice deficient in T lymphocytes exhibit cognitive impairment, while passive transfer of mature T cells reversed this effect [62]. Thus, when SCID BALB/c/OLA mice deficient in mature T cells were compared to wild-type (WT) mice by subjecting them to the Morris water maze behavioral test, a visual-spatial learning/memory task, a remarkable impairment of spatial memory was revealed in the mice lacking adaptive immunity. This effect was reversed by T-cell supplementation. In general, T cells interact with antigen-presenting cells (APC) at the site where the T-cell receptor (TCR) is triggered by its antigen ligand peptide presented by the MHC within the APC membrane [63, 64]. It has been previously reported that cellular targets of T cells with greatly reduced expression of MHC class I molecules still retain the capacity to form synapses [65]. Evidence suggests that barely detectable levels of MHC molecules in neurons enable the interaction with T cells [66, 67]. This may result in the regulation of synaptic plasticity during development and after injury, as neuronal MHC expression can mediate T-cell responses [68].

T cells are critical contributors to the immune response in many disease states, including after an ischemic insult. Several studies using a mouse model of permanent middle cerebral artery occlusion (MCAO) show that their infiltration into the brain parenchyma peaks 3–7 days after insult, but that they persist for much longer in the ischemic mouse brain [69, 70], suggesting a long-term T-cell response and their involvement in recovery processes [71]. T cells in the brain parenchyma expressing activation markers persist until at least day 28 in stroke mice [72, 73]. Persistent T-cell accumulation has also been detected in poststroke patients, more than 1 month after the ischemic event [74].

Exactly how T cells influence injury and whether this is mediated by interactions with neurons after ischemia are poorly understood. It has been shown that early detrimental T-cell effects in experimental stroke are not related to adaptive immunity, as TCR-transgenic mice and mice lacking costimulatory TCR signals are fully susceptible to tMCAO [75]. Nevertheless, other disease models have demonstrated the importance of direct interactions between T cells and neurons [76–78]. Indeed, MHC class I expression in the brain is reportedly induced at least 24 hours (h) following an ischemic injury, mainly in neurons [79, 80]. This is further supported by the finding that MHC class I proteins are expressed by neurons and are closely associated with synaptic markers in the healthy brain, as they have been found at synapses both in the presynaptic terminal associated with synaptic vesicles and in the postsynaptic spine or dendritic shaft associated with the postsynaptic density [67, 81, 82]. Therefore, MHC class I molecules may be abundant enough on neurons to allow neuro-immune synapse formation (Fig. 1) [66, 82, 83]. Indeed, CD8 T cells form immune synapses and actively engage virus-infected neurons during acute infection in a Theiler’s murine encephalomyelitis virus model of multiple sclerosis [83]. Although potentially different mechanisms are at play, patterns from acute inflammation may help to understand the interplay of different cellular subtypes at the synapse in sterile inflammation.

There is increasing evidence of the importance of the adaptive immune system in facilitating/impeding functional recovery after stroke. As mentioned above, T cells (CD4 + and CD8 +) remain activated in the late stage of ischemic stroke coinciding with neuronal plasticity and the recovery period [84]. In fact, long-term activation of T cells after stroke, together with their known role in the regulation of healthy neuronal circuits [73], suggests that T cells are potential mediators of beneficial effects in plasticity, enhancing recovery for an extended period after stroke [85, 86].

B cells constitute another adaptive immune cell subset with the ability to produce neurotrophins to support neuronal survival and plasticity [87, 88]. They have been shown to be the most abundant infiltrating lymphocytes in the developing brain, as they play a key role in brain development during the neonatal period [89]. B cells are abundant in the parenchyma 48 h after stroke onset and are recruited by the expression of CXCL13 on the cerebrovascular endothelium, a chemokine that specifically recruits B cells during inflammation [90, 91]. B cells are important components of the adaptive immune system that promote neurological recovery after stroke by participating in processes such as neurogenesis, resulting in long-term recovery of motor function and improvement of spatial memory [90, 92]. They migrate to remote regions of the brain that are undergoing poststroke plasticity and are associated with neurogenesis distant to the infarct epicenter and respond to local signals that enhance their neurotrophic capacities to promote neuroplasticity [93, 94]. Exposure of mixed cortical cells to 2 h of oxygen–glucose deprivation (OGD) in culture followed directly by the addition of naïve B cells reduces neuronal death (as detected by a neuronal marker, microtubule-associated protein) and loss of dendritic arborization [94]. The protective functions of B cells in ischemic stroke include direct synaptic modulation, thereby regulating motor and cognitive functions, which increases in these cellular processes requiring the active presence of B cells after stroke [94].

On the other hand, Doyle et al. show that the increased B-cell influx is seen 1 to 2 weeks after MCAO. Thereafter, their number continues to increase at least until 10 weeks after stroke. They postulate that this accumulation of B cells is responsible for delayed cognitive decline after stroke [95]. The role of B cells in cerebral ischemia remains inconclusive and requires further investigation. However, there is evidence for a prominent role of B cells in promoting stroke recovery, especially after the acute/subacute phase of stroke [94]. In summary, it is clear that infiltrated circulating components of the adaptive immune system have a role not limited to secondary inflammation after stroke with the potential to affect synaptic performance and recovery.

## Neuro(adaptive)immunological Synapses with Indirect Neural Effects

Cytokines are small pleiotropic signaling proteins secreted by a variety of immunocompetent cells, including monocytes, macrophages, lymphocytes, and vascular endothelial cells. The cytokine family includes interleukins, chemokines, tumor necrosis factors, interferons, growth, and cell-stimulating factors as well as neurotrophins [96]. Cytokines are highly involved in neurodevelopmental processes that control neuronal migration and synaptogenesis, shaping to a large extent neural activity [97, 98]. Variable cytokines have been observed at mature synapses, as some are involved in direct basal synaptic transmission, for example, by affecting the surface receptor expression of glutamatergic receptors, tipping the excitatory/inhibitory balance to one side or the other [99–101].

After stroke, the inflammatory niche of the ischemic area contains a diverse array of cytokines that regulate the innate and adaptive immune response and contribute significantly to disease progression [102]. Microglia, astrocytes, endothelial cells, and neurons in the brain are all capable of secreting pro- and anti-inflammatory cytokines [103]. In general, increased secretion of pro-inflammatory cytokines and decreased levels of anti-inflammatory cytokines correlate with worse outcome in experimental stroke models [104]. As mentioned above, recent evidence suggests that B cells are not detrimental to neurons after stroke but rather facilitate functional recovery [105]. One possible mechanism by which they do this is by producing brain-derived neurotrophic factor (BDNF), nerve growth factor (NGF), and IL-10, all of which are necessary for the maintenance of mature neurons and neuronal protection [87, 106, 107].

We have previously demonstrated a key protective role of the anti-inflammatory cytokine IL-10 after ischemic insult [108]. We were able to show that IL-10 overexpression in T cells modulates microglial genes involved in synaptic pruning and downregulates the complement system in microglia, thereby promoting amelioration of functional deficits assessed by forelimb asymmetry [108, 109]. This suggests a role for cytokine secretion in improving functional deficits in late recovery after stroke [109]. In the ischemic brain, sources of IL-10 include regulatory T cells, macrophages, and microglia, all of which are at in close proximity to the ischemic zone and come into close apposition with neurons [108].

Another family of cytokines with immunomodulatory functions that tightly regulate synaptic plasticity in the healthy CNS are type I and type II interferons [110]. In stroke, interferon (IFN)-γ released from Th1 cells has been shown to worsen disease outcome [111–113]. Moreover, spleen-derived IFN-γ is indirectly involved in stroke-induced neurodegeneration through downstream activation of macrophages/microglia, and its inhibition reduced neurodegeneration after permanent MCAO in rats [114]. On the other hand, IFN-β, which belongs to the type I interferon family, promotes an anti-inflammatory milieu by reducing neuronal cell death, regulating peripheral immune cell infiltration, and increasing functional recovery after stroke [115–117]. IFN-β administration reduced immune cell infiltration into the ischemic mouse brain [118]. Type I IFNs activate microglia and induce synapse elimination in a mouse model of Alzheimer’s disease, while IFN blockade restores synaptic loss in vivo [119]. Comparisons between Alzheimer’s disease and ischemic stroke in fine-grained gene ontology resources revealed major similarities between the two disorders in synaptic pathways [120]. Nevertheless, there is still much to be learned about how immune-secreted factors influence synaptic loss in stroke.

A recent focus is the study of heterogeneous, double membrane-enclosed structures termed extracellular vesicles (EVs). Similar to cytokines, EVs are ubiquitous conveyors of intercellular messages that function as intercellular immune mediators [121]. Since their discovery, EVs produced by glial cells, including astrocytes, microglia, NG2 glia, oligodendrocytes, radial glial cells, and ependymal cells, have been shown to play an important role in immune signaling during synaptic plasticity [122]. EVs derived from astrocytes are internalized by neurons and contribute to synaptic homeostasis [123]. Recently, a study compared the proteomic profile of EVs derived from astrocytes stimulated with either ATP or the anti-inflammatory cytokine IL-10 and showed that they are enriched in proteins involved in neurite outgrowth, axonal guidance, synaptogenesis, and synaptic long-term potentiation when compared to unstimulated astrocyte EVs, confirming their role in transsynaptic communication [124]. Accumulating evidence indicates that EVs regulate ischemia-related pathological processes, including fundamental processes of innate and adaptive immunity, such as inflammation, antigen presentation, and B- and T-cell development and activation [121, 125]. By isolating EVs directly from mouse brain tissue, we have shown that microglia cells release relatively higher amounts of EVs under physiological conditions, whereas astrocytes release more EVs 24 h after stroke [126]. EVs have been shown to affect the function of immune synapses which share properties with neuronal synapses [127]. In this sense, non-neuronal cells (such as T cells and microglia) indirectly modulate synaptic function through the release of EVs.

## Neuro(innate)immunological Synapses with Direct Neural Effects

Microglia are the main innate resident immune cells of the brain parenchyma and major participants in synaptic plasticity under homeostatic conditions, directly interacting with presynaptic or postsynaptic neuronal components [128–130] constituting the quadripartite neuroimmunological synapse [18]. In addition, microglia form physical contacts with several other neuronal compartments such as axon initial segments, the nodes of Ranvier, and neuronal somata [43, 131]. This direct interaction is one way by which microglia interact with and influence synapses and other sites on neurons, potentially allowing for fine-tuned regulatory actions. During development, microglia rid the brain of both inhibitory and excitatory synapses, while microglial depletion during this period results in an overabundance of synapses that are abnormally regulated, leading to behavioral abnormalities in mice [132]. It should be noted, however, that somatic interactions between microglia and neurons (i.e., those mediated by somatic purinergic junctions) are likely to occur at earlier stages of development, before the development of synapses [43, 133]. In the healthy brain, microglia continuously survey the surrounding microenvironment and interact with synapses to ensure the proper formation of neuronal circuits while also performing other surveillance functions such as synaptic phagocytosis or secretion of soluble factors [129, 134–136]. Similarly, astrocytes structurally compose the tripartite synapse as their processes envelop synapses and are tightly associated with the regulation of synapse formation and function [137]. During development, astrocytes are actively involved in synapse formation from the early stages of synapse formation and are indirectly involved in neuronal plasticity [138]. Furthermore, in the mature brain, astrocyte processes wrap presynaptic and postsynaptic terminals and limit neurotransmitter diffusion by the uptake of neurotransmitters from the synaptic cleft.

Resident microglia are essentially the innate scavenger cells involved in tissue degeneration and repair [139]. Primed microglia adopt variable phenotypes and functions in a spatiotemporal manner after an ischemic insult [140]. They are detected within the first hours (6 h) after stroke and secrete chemokines that further promote the infiltration of peripheral immune cells [141]. During disease states, microglia may act as local APCs to initiate, regulate, and maintain an immune response [142, 143]. In stroke, resident microglia contribute to the pathogenic cascade following an ischemic insult by secreting factors involved in secondary infarct expansion [144, 145]. This is evidenced by the rapidity with which microglia can alter neuronal activity and modulate synaptic function, thereby supporting recovery from stroke injury [146]. In vivo two-photon imaging has shown that synapses in ischemic regions exhibit enhanced turnover rates upon contact with microglia, suggesting that microglia are destined to prune synapses after stroke [129]. The efficient pruning of synapses is thought to promote tissue reconstruction and neuronal network reorganization after stroke [147]. One day after transient stroke, microglia-specific deletion of brain-derived neurotrophic factor (BDNF) influences glutamatergic and GABAergic synapses, thereby rescuing neuronal cell death and maintaining synaptic integrity [148]. In this study, the authors show that the loss of microglia activation in a model of conditional knockout mouse where BDNF expression is selectively inhibited in microglia suggests that the prevention of microglia BDNF release preserves synapses and prevents alterations in microglia morphology [148]. The development of neuroplasticity is spatiotemporally consistent with microglial activation, indicating that microglia have a profound impact on postischemic neuroplasticity, making them a key therapeutic target for poststroke rehabilitation [149].

However, microglial contributions to stroke are highly complex and cannot be deduced simply by studying microglia-synapse interactions. Recent studies show that the net effect of microglia on stroke outcome, at least in acute stages, may be protective, whereas dysfunctional microglia may exert multiple deleterious effects at different stages after stroke. This diversity of microglial contributions has important implications for the development of drugs that target microglial dysfunction to selectively block harmful contributions without interfering with inherently protective microglial responses. Indeed, blockade of microglial proliferation and microglial depletion/blockade with the CSF1R inhibitors (PLX3397, PLX5622, or ki20227) or anti-CSF1R antibodies (AFS98) exacerbate brain injury after transient or permanent MCAO and global cerebral ischemia in both young and aged animals [139, 150–153]. Acute blockade of microglial P2Y12 receptors also leads to pathological neuronal network activity and greater brain injury after stroke, in part via somatic purinergic junctions, while also impairing vasodilation, increasing hypoperfusion and BBB injury in non-stroke models [154–158]. Therefore, it will be important to study how changes in microglial phenotypes may affect their communication with different neuronal compartments and other cell types and how these interactions change in disease states. With respect to NI-related changes, it is also likely that systemic inflammatory mediators and immune cells recruited to the brain after stroke could alter microglial phenotypes and thus shape the direct interactions formed between microglia and synapses or other cells. For example, early activation of microglia in response to stroke is differentially regulated by distinct T-cell subpopulations, and the formation of pro-regenerative microglial phenotypes could occur via IL-10-mediated mechanisms [159]. Thus, the formation and molecular architecture of NI under different inflammatory and disease states appear important to define.

Failure of neuronal regeneration may be related to inadequate clearance of cellular debris by scavenging microglia [160]. However, recent studies have also suggested that dysregulated microglia/macrophages damage synapses in Alzheimer’s disease and viable neurons in stroke [161–164]. Shi et al. demonstrated that endogenous microglia react to neuronal tissue damage by engulfing synapses in the area of active gliosis, thereby impeding brain repair. They demonstrated that a specific inhibition of the phagocytic reactive microgliosis maturation reduced synapse loss and improved neurobehavioral outcomes [165]. The optimal amount and temporal activity of synaptic engulfment seem to be a key determinant between degeneration and regeneration. Interestingly, the phagocytic biomarker Mac-2 peaks on day 3 in microglia and steadily decreases over the next 14 days, unlike Mac-2 expression in astrocytes, which is elevated from day 1 to day 14 following middle cerebral artery occlusion [165]. Since microglial activation coincides with neuroplasticity 3–4 weeks after stroke [140], they must influence synaptic recovery in alternative ways not involving their phagocytic behavior.

Both microglia and astrocytes perform classical innate immune system functions in the brain [166, 167]. The innate immune functions of astrocytes have been largely ignored until recently due to the widespread belief that microglia are the sole effector immune cells in the CNS [168, 169]. Astrocytes influence synaptic damage or recovery at different stages of ischemic stroke through numerous proposed pathways such as glutamate clearance or secretion of factors such as TNF-α or thrombospondin, which are involved in synapse formation and function [137]. They play an important role in glutamate regulation and uptake, which influences poststroke excitotoxicity and reduces hyperexcitability [170]. The astrocyte-secreted enzyme autotaxin is elevated after stroke, leading to cortical hyperexcitability [171]. Astrocytes also share complex phagocytic functions with microglia, particularly in the clearance of damaged neurons, which is mediated in part via the receptor tyrosine kinase *Mertk* [172]. In this context, it remains important to identify the key regulators of phagocytosis by these cells with respect to different neuronal compartments and the molecular assemblies that block or promote engulfment of synapses or other structures.

Another member of the innate immune system that is one of the first effectors at sites of inflammation is natural killer (NK) cells [173]. Ischemic neurons secrete CX3CL1 which recruits NK cells within 24 h after stroke. Due to their proximity to neurons in the peri-infarct area, recruited NK cells tend to form complexes with neurons that structurally resemble the immune synapse [174]. NK cell-mediated exacerbation of damage occurs rapidly after ischemia. NK cells tend to increase synaptic excitatory transmission, leading to neuronal hyperexcitability. Interestingly, this finding implies that the role of NK cells is not only limited to poststroke inflammation but may also have direct effects on synaptic excitability.

## Neuro(innate)immunological Synapses with Indirect Neural Effect

Innate immune cells are an identified source of neurotransmitters that allow bidirectional crosstalk between the nervous and immune systems [175]. They also secrete soluble mediators that modulate homeostatic plasticity in both excitatory and inhibitory neurons [176–179]. Developmentally active pathways that are associated with synaptic pruning or elimination, such as those involving the complement system, may be reactivated in neurodegenerative diseases [180].

When activated by inflammatory stimuli, neutrophils produce neurotransmitters like acetylcholine and catecholamines that can feed back to the original neuronal network, forming a loop that ultimately signals back to neutrophils [175, 181]. At the same time, neutrophil pro-inflammatory activation after stroke is associated with increased infarct size, increased BBB disruption, and worse neurological outcomes in general [182].

In addition to direct interactions with neurons, microglia can influence synapses in an indirect manner. As mentioned above, perturbation of microglial homeostasis leads to increased cytokine secretion as part of their immune response, which either facilitates recovery or promotes secondary damage [183]. Distinguishing between extracellular molecular and intracellular key signals that control microglial phenotypes is necessary to identify an appropriate clinical treatment regimen. This is because the roles of pro- and anti-inflammatory microglial functions are not necessarily destructive or beneficial, respectively. Classically activated microglia may contribute to synaptic remodeling after stroke, whereas alternatively activated microglia may play a role in modulating synaptic plasticity through the inflammatory cytokine tumor necrosis factor (TNF-α), which has also been studied in nerve injury and ischemic stroke [184, 185]. One example is the microglia-derived TNF-associated weak inducer of apoptosis (TWEAK) and its neuronal receptor Fn14, which interact to regulate synaptic function in stroke [186]. TWEAK and Fn14 are upregulated in the CNS after ischemic stroke in humans and mice [187–189]. They are synthesized and released by various cell types in response to injury [190]. Upon activation, TWEAK acutely attenuates basal synaptic transmission and plasticity while altering the phosphorylation state of pre- and postsynaptic proteins in adult mouse hippocampal slices [191]. Nagy et al. showed that depletion or pharmacological inhibition of TWEAK/Fn14 signaling enhances synaptic transmission and plasticity [191].

Microglia respond to fluctuations in activity by engaging classical inflammatory signaling cascades to influence synaptic activity [97]. The anti-inflammatory cytokine IL-4 is another factor secreted by alternatively activated microglia, as reported by Liu et al. who demonstrated that IL-4 secreted by microglia reduced infarct size after ischemic stroke and improved long-term functional recovery [192]. Conversely, stroke mice that lost IL-4 exhibit intrinsic hyperexcitability and enhanced excitatory synaptic transmission [193].

Toll-like receptors (TLRs) are other major regulators of innate immunity that play an integral role in the activation of the inflammatory response during infection with important modulatory roles in stroke [194]. Microglia express TLR2 in response to ischemia, as shown in rats in vivo and acutely (6 h after exposure to ischemic conditions) in vitro [195]. In this case, microglia exacerbate neuronal damage by secreting toxic cytokines such as IL-23 and IL-17. Knockdown of TLR2 in microglia actually alleviates neuronal damage [195]. This mechanism of neuronal injury in stroke requires further examination. In addition, the effects of TLR ligands on microglia and neuronal network function have been explored in rat organotypic hippocampal slice cultures [196]. Notably, activation of TLR3, which is predominantly expressed on microglial cells, induces neuroprotective properties in microglia under noninfectious conditions in vivo [197, 198]. Systemic administration of a TLR3 agonist results in increased IFN-β-mediated hippocampal excitability [199]. However, how microglia TLRs induce neuronal network dysfunction requires further studies in stroke models.

The complement system is another key innate defense system that is rapidly triggered upon injury and shows extensive crosstalk with TLRs [200]. Some components of the system have been shown to play a beneficial role in synapse elimination during development [201]. Complement components have been shown to be elevated in the serum of patients after stroke during the subacute phase and remain chronically elevated [202, 203]. Although complement-induced neuroinflammation begins acutely, it has been shown to contribute to chronic inflammation, too. This chronic response results in complement-dependent synaptic degeneration and poor cognitive recovery [204]. There is emerging evidence for a role of the complement system in synapse repair and plasticity after ischemic stroke that begins during the acute phase and continues chronically (at least 30 days after stroke) [204]. This demonstrated using the complement (C3) inhibitor B4Crry, which reduced microgliosis and synaptic engulfment, increased synaptic density, and improved cognitive outcome after stroke. Complement activation and opsonization in turn affect microglial phagocytosis of synapses, leading to synaptic loss and limiting cognitive recovery in stroke mice [204].

Ischemic brain tissue accumulates a small subset of T cells called gamma-delta (γδ) T cells, which contribute to the pro-inflammatory repertoire by promoting neutrophil infiltration [205]. γδT cells produce the majority of IL-17A in the acute phase (3 days following stroke), while the majority of IL-17A in the chronic phase (28 days later) is produced by astrocytes, and have different roles in recovery at different temporal stages [206]. Under neuroinflammatory conditions, IL-17 A is upregulated in reactive astrocytes [207, 208]. This elevation of astrocytic IL-17A supports spontaneous recovery after stroke and promotes synaptogenesis [209]. IL-17A promotes the survival of neural progenitor cells and ensures their proper integration into the striatal network resulting in improved long-term functional recovery, as also demonstrated on the behavioral level. Thus, IL-17A shows a biphasic temporal expression with opposing roles, both deleterious and beneficial effects in neuroinflammation, linking innate and adaptive immunity [207, 210, 211]. This supports long-term recovery and functional integration of newly formed neurons, restoring damaged neuronal networks based on the recent study showing that the targeted expression of the transcription factor NeuroD1 in microglia/macrophages present in the peri-infarct area can convert this cell population into striatal projection neurons in ischemic regions of tMCAO mice [212].

## Conclusion

The crossroads where neurotransmitters and classical immune-related molecules meet are at the level of the neuroimmunological synapse, a “hybrid junction” that modulates synaptic plasticity and allows direct interactions between the two systems [19, 213, 214]. The concept of the immunological synapse has been attributed mainly to cells of the adaptive immune system, T and B cells, but now includes interactions with innate immune cells such as natural killer (NK) cells and, more recently, phagocytes [39, 215–218]. They share features with neuronal synapses such as the presence of a synaptic cleft, adhesion molecules, and polarity [219]. Studies using biophysical analysis and two-photon microscopy have confirmed the presence of such synapses with clear and robust interactions. The majority of described immune synapse interactions revolve around microglial cells. The investigative focus should shift towards understanding the modes of interaction of different components of the immune system with neuronal synapses to support recovery after ischemic injury.

## Data Availability

No datasets were generated or analysed during the current study.
